# Carbon dioxide and nitrate co-electroreduction to urea on CuO_*x*_ZnO_*y*_

**DOI:** 10.1038/s42004-023-01001-5

**Published:** 2023-09-19

**Authors:** Dimitra Anastasiadou, Bianca Ligt, Yunyang He, Rim C. J. van de Poll, Jérôme F. M. Simons, Marta Costa Figueiredo

**Affiliations:** 1 Department of Chemical Engineering and Chemistry, Eindhoven University of Technology, PO Box 513, Eindhoven, 5600 MB the Netherlands; 2 Eindhoven Institute of Renewable Energy Systems (EIRES), Eindhoven University of Technology, PO Box 513, Eindhoven, 5600 MB the Netherlands

**Keywords:** Electrocatalysis, Energy, Sustainability, Electrocatalysis, Materials chemistry

## Abstract

Urea is a commonly used nitrogen fertiliser synthesised from ammonia and carbon dioxide using thermal catalysis. This process results in high carbon dioxide emissions associated with the required amounts of ammonia. Electrocatalysis provides an alternative method to urea production with reduced carbon emissions while utilising waste products like nitrate. This manuscript reports on urea synthesis from the electroreduction of nitrate and carbon dioxide using CuO_x_ZnO_y_ electrodes under mild conditions. Catalysts with different ratios of CuO and ZnO, synthesised via flame spray pyrolysis, were explored for the reaction. The results revealed that all the CuO_x_ZnO_y_ electrocatalyst compositions produce urea, but the efficiency strongly depends on the metal ratio composition of the catalysts. The CuO_50_ZnO_50_ composition had the best performance in terms of selectivity (41% at −0.8 V vs RHE) and activity (0.27 mA/cm^2^ at −0.8 V vs RHE) towards urea production. Thus, this material is one of the most efficient electrocatalysts for urea production reported so far. This study systematically evaluates bimetallic catalysts with varying compositions for urea synthesis from carbon dioxide and nitrate.

## Abstract (web summary)

The simultaneous electroreduction of carbon dioxide and nitrate is a promising and environmentally benign route to urea production, but achieving high selectivity for urea electrosynthesis via this route remains challenging. Here, CuO_x_ZnO_y_ electrodes are shown to enable the efficient and selective production of urea under mild conditions, with the efficiency found to strongly depend on the metal ratio within the catalyst composition.

## Introduction

The use of urea in the chemical industry is vast, ranging from fertilisers to pesticides and medicines. Urea-based fertilisers account for 70% of global nitrogen-containing fertilisers. Currently, urea is produced by the reaction of liquid NH_3_ and CO_2_^1^ in a process known as the Bosch-Meiser. The required ammonia is synthesised by the Haber-Bosch process, which relies on fossil fuel resources and is known as one of the most significant contributors to CO_2_ emissions. As a consequence, Bosch-Meiser suffers from high energy input (150–200 °C and 100–200 atm) and high CO_2_ emissions. Therefore, much effort has been dedicated to exploring green and sustainable strategies for urea synthesis^2,3^. The synthesis of urea with electrochemical methods has increased attention from the scientific community since it can help reduce the level of CO_2_ emissions currently associated with urea production^4^. Various nitrogen sources are being explored to realise the C-N bond formation via CO_2_ co-electroreduction, including nitrate (NO_3_^−^), nitrite (NO_2_^−^), NO, and N_2_^5–11^. Nitrate is a particularly attractive nitrogen feedstock because of its high solubility in water and low dissociation energy. Moreover, NO_3_^−^ pollution in water is problematic as it can lead to severe human and environmental problems, such as cancer or eutrophication. Thus, this approach can mitigate NO_3_^−^ contamination in water by using nitrate-rich streams or effluents.

Shibata et al.^12^. first reported urea electrosynthesis by converting CO_2_ and NO_3_^−^/ NO_2_^−^ at mild conditions. Amongst the various metals tested, Cu and Zn showed promising Faradaic efficiency to urea (12 and 29%, respectively) at ca. −0.9 V vs RHE. Several studies were later published exploring catalysts such as In(OH)_3_ or ZnO^9,13^. In(OH)_3_ showed a remarkable Faradaic efficiency of 53% for urea synthesis from NO_3_^–^ and CO_2_^13^. The activity of this catalyst has been attributed to the key step of coupling *NO_2_ and *CO_2_ on the (100) surface of the catalyst. At the same time, its semiconducting nature suppressed H_2_ generation. On the other hand, the study of ZnO porous nanosheets revealed that the presence of surface oxygen vacancies was beneficial for the reaction and efficiencies of 23.3% at −0.79 V vs RHE were obtained. In that study, urea was obtained from NO_2_^-^ and CO_2_ and COOH* was identified as the intermediate required for the C-N formation^9^.

One strategy to optimise the selectivity of electrocatalysts is the use of bimetallic materials. Combining two metals with different properties can change the adsorption energy and intramolecular bond energy in adsorbed reactants and intermediates. In addition, strain effects can also occur when one of the metal atoms is forced to adopt positions different from the equilibrium position in the bulk materials resulting in the modification of the surface electronic structure^14–16^. Using bimetallic materials is unsurprising for urea electrosynthesis as the catalysts should provide optimal affinities for the required intermediates from the two parallel reactions (CO_2_ and NO_3_^−^ electrochemical reduction) with significantly different surface requirements. Copper has attracted the attention of researchers for the electrosynthesis of urea^17,18^ due to its ability to reduce CO_2_^19^ and NO_3_^−^^20,21^ separately. A few Cu-based bimetallic catalysts have been reported^22,23^. For example, Cu@Zn core-shell nanowires structures produced urea from CO_2_ and NO_3_^−^ with 9.28% Faradaic efficiency at −1.02 V vs RHE^22^. The authors identified *CO and *NH_2_ as critical intermediates for the C–N bond formation with these catalysts. Despite the encouraging results, achieving a highly selective urea synthesis via simultaneous electroreduction of CO_2_ and NO_3_^-^ remains challenging. Moreover, in the reported work, no attention was paid to the metal ratios of the electrocatalysts and their influence on the reaction efficiency.

In this work, we investigated CuO_*x*_ZnO_*y*_ catalysts for the electrosynthesis of urea by coupling the CO_2_ and NO_3_^−^ reduction reactions. The effect of the different ratios of CuO_*x*_ZnO_*y*_ on the reaction efficiency is explored. All the catalysts that contain both metal oxides produced urea, but a strong dependency on the amount of ZnO was found for the amount of urea produced. Within the studied catalysts, CuO_50_ZnO_50_ exhibited the highest Faradaic efficiency (41% at −0.8 V vs RHE) in an electrolyte with continuous CO_2_ flow. This work provides an effective strategy for designing and optimising bimetallic catalysts with superior properties to achieve high selectivity for urea electrosynthesis.

## Results and discussion

### Catalyst characterisation

Catalysts were prepared with varying ratios of CuO and ZnO using flame spray pyrolysis (FSP) (Fig. S1). The different catalyst materials are named CuO, ZnO and CuO_*x*_ZnO_*y*_, where *x* and *y* represent the mole percentage of CuO and ZnO, respectively. The materials were characterised by using transmission electron microscopy (TEM), scanning electron microscopy (SEM) with energy-dispersive X-ray spectroscopy (EDX), wide-angle X-ray scattering (WAXS) and X-ray photoelectron spectroscopy (XPS).

**Fig. 1:**
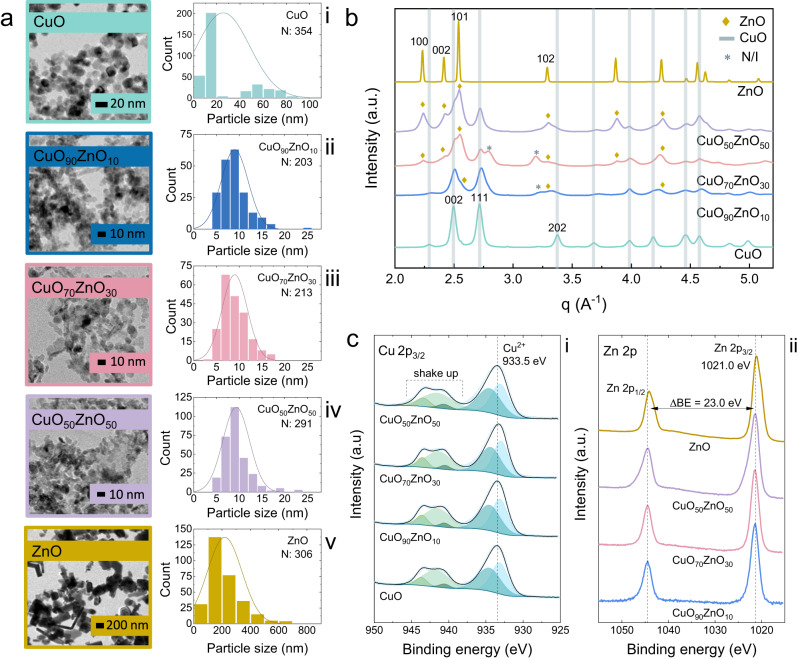
Structural and compositional characterisation of CuO_*x*_ZnO_*y*_ catalysts. **a** TEM images and particle size distributions of CuO_*x*_ZnO_*y*_ catalysts, **b** WAXS patterns of CuO_*x*_ZnO_*y*_ catalysts and (**c**) XPS spectra of the CuO_*x*_ZnO_*y*_ catalysts (**i**) Cu 2p_3/2_ and (**ii**) Zn 2p.

In Fig. 1a (Supplementary Data 1), the TEM images of the catalysts are presented. The ZnO particles are the largest in the group, with an average length of 0.2 μm. Moreover, they showed a polydisperse morphology, mostly with elongated rod structures. The pure CuO showed a bimodal distribution of particles with an average size of 15 nm and bigger aggregates with an average size of 60 nm. (Fig. 1a-I, Supplementary Data 1). The addition of ZnO seems to prohibit the aggregate formation as all the bimetallic CuO_*x*_ZnO_*y*_ materials have a 9 nm average particle size. The diffraction patterns of the particles were obtained using the Fast Fourier Transform of the image, and information about the crystallographic plane of the particle edges was found by matching the interplanar spacing (d). For ZnO, the average value of the interplanar spacing is 0.283 nm, which correlates to the {100} planes of the hexagonal ZnO structure (Fig. S2). These results agree with previous reports on flame spray pyrolysis synthesis of ZnO^24–26^. For the CuO, the measured value is 0.256 nm. This value is close to the lattice spacing of the {111} planes in the bulk of a monoclinic CuO^27,28^. In the mixed oxides, CuO_90_ZnO_10,_ CuO_70_ZnO_30,_ the measured lattice spacing is 0.265 nm. This value is slightly higher than the value measured for CuO, suggesting some insertion of Zn within the CuO lattice. For CuO_50_ZnO_50_, lattice spacings of 0.265 and 0.296 nm were measured; both values are higher than those from CuO and ZnO. It has been reported that Zn-doping of the CuO and Cu-doping of the ZnO structure can occur^27^. Albeit the Zn^2+^ and Cu^2+^ ionic radii are nearly the same size, significant doping may lead to lattice constant expansion. This suggests that, for the catalysts with lower amounts of ZnO (CuO_90_ZnO_10,_ CuO_70_ZnO_30_), the increased lattice spacing is due to the doping of the CuO structure with Zn. When the CuO and ZnO amounts are the same, doping of the ZnO structures also occurs, as evidenced by two values for the lattice spacing on the CuO_50_ZnO_50_ catalysts.

The WAXS patterns of the CuO_*x*_ZnO_*y*_ materials shown in Fig. 1b (Supplementary Data 1) strongly demonstrate their polycrystalline nature. The diffractograms show peaks for ZnO characteristic of a hexagonal structure (PDF-00-065-0726, Fig. S3, Supplementary Data 2) at q positions of 2.23 A^−1^ (100), 2.41 A^−1^ (002), 2.54 A^−1^ (101), 3.29 A^−1^ (102) and 2θ positions of 32.51° (100), 35.18° (002), 37.00° (101) and 42.22° (102) (Fig. S3). For CuO, the peaks are observed at 2.50 A^−1^ (002), 2.71 A^−1^ (111), and 3.37 A^−1^ (202) and can be attributed to the monoclinic structure (PDF-00-005-0661, Fig. S3) with 2θ positions of 35.59° (002), 38.81° (111) and 48.82° (202) (Fig. S3, Supplementary Data 2)^24^. For the CuO_*x*_ZnO_*y*_ bimetallic materials, the XRD patterns show features of both CuO and ZnO phases. Specifically, the diffraction peaks of CuO (002) and (111) are present in all samples, but the CuO (111) becomes less pronounced with the increase of ZnO. The ZnO (100), (002), and (101) peaks increase in intensity with a higher amount of Zn present. A small diffraction feature is observed at 40.17° in the CuO_70_ZnO_30_ and CuO_90_ZnO_10_ diffractogram. This peak could not be attributed to the CuO and ZnO individual structures. The origin of this peak can tentatively be attributed to Cu suboxides, but further investigation is needed to identify this phase^29–31^.

The surface electronic states and the chemical composition of the materials were investigated using XPS. In Fig. 1c (Supplementary Data 1), the Cu 2p_2/3_ and Zn 2p spectra of all the samples are plotted. The Cu 2p_2/3_ spectra show a peak at 933.5 eV, corresponding to the oxidation state of Cu^2+^^32^. The shake-up peaks between 940 and 944 eV further confirm this observation^32^. The Cu^2+^ oxidation state of the materials is also notable from the shape and position of the Auger peak in Fig. S4a (Supplementary Data 2). These results revealed that in all synthesised materials, the electronic state of Cu (CuO) is independent of the amount of ZnO. The Zn 2p spectra show a difference of 23 eV between the Zn 2p_1/2_ and Zn 2p_3/2_ for all the samples, which is characteristic of ZnO^33^. However, when comparing the Zn 2p_3/2_ peak of ZnO (1021 eV) with the CuO_*x*_ZnO_*y*_ samples, a small swift towards lower binding energy is visible that can be assigned to their large particle size difference^34^. Since the Zn 2p_3/2_ states for Zn metal and Zn^2+^ peak overlap, it is preferred to use the Auger parameter to identify the oxidation state of Zn (Fig. S4b, Supplementary Data 2). From the Zn LMM spectra, it is visible that the oxidation state is Zn^2+^ for all samples^34–36^.

**Fig. 2:**
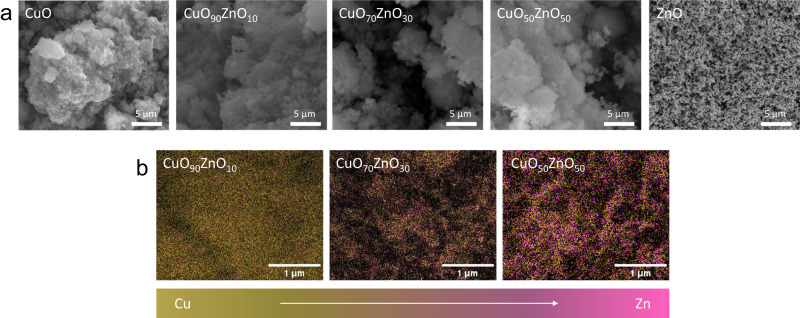
Electron microscopy characterisation of the CuO_*x*_ZnO_*y*_ catalysts. **a** SEM images of the CuO_*x*_ZnO_y_ catalysts, (**b**) SEM-EDX images of the CuO_90_ZnO_10_, CuO_70_ZnO_30_, and CuO_50_ZnO_50_ catalysts.

The composition of the samples was further verified by inductive coupled plasma-optical emission spectrometry (ICP-OES) elemental analysis. The metallic compositions were obtained from the ICP-OES and XPS and are compared in Table S1. The results showed that similar metal ratios were obtained by both techniques. SEM-EDX was performed as a bulk analysis technique to assess the composition and homogeneity of the samples (Fig. 2). The CuO_*x*_ZnO_*y*_ composite materials showed a homogeneous distribution of Cu and Zn over each catalyst. The atomic percentages obtained from the SEM-EDX analysis showed similar values to the ones from ICP-OES and XPS.

### Electrochemical measurements

**Fig. 3:**
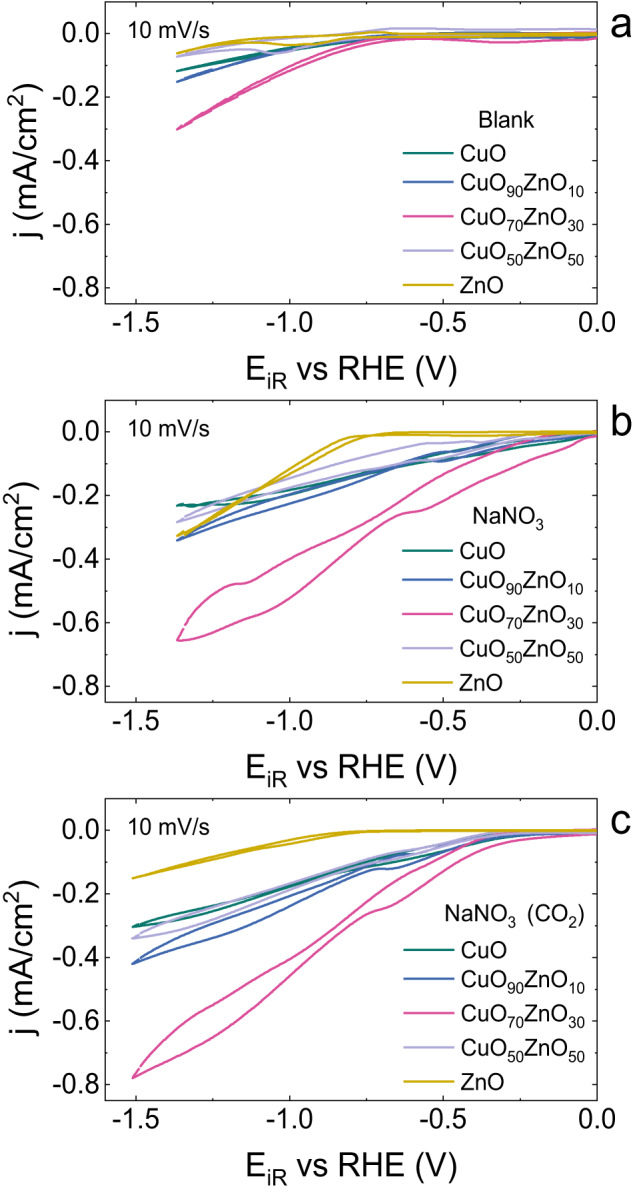
Cyclic voltammograms of the CuO_*x*_ZnO_*y*_ catalysts. **a** cyclic voltammograms of the CuO_*x*_ZnO_*y*_ catalysts in 0.1 M Na_2_SO_4_ (blank), (**b**) cyclic voltammograms of the CuO_*x*_ZnO_*y*_ catalysts in 0.1 M Na_2_SO_4_ and 0.1 M NaNO_3_ and (**c**) cyclic voltammograms of the CuO_*x*_ZnO_*y*_ catalysts in 0.1 M Na_2_SO_4_ and 0.1 M NaNO_3_ with CO_2._ Scan rate: 10 mV/s.

To characterise the prepared catalysts electrochemically, cyclic voltammograms (CVs) were recorded in the supporting electrolyte 0.1 M Na_2_SO_4_ at potentials between 0.6 V and –1.4 V vs RHE at a scan rate of 10 mV/s (Figs. 3a, S5, Supplementary Data 1, 2). The iR corrected CVs, normalised for the calculated electrochemical surface area, show that for potentials between 0.5 and −0.5 V vs RHE, only minor redox features related to the metal redox states are observed. The reductive currents increase significantly at potentials more negative than ca. −0.75 V vs RHE. Since no reactants are present in those conditions, the current can be attributed to the hydrogen evolution reaction (HER). The HER currents increase with increasing ZnO content in the catalysts up to CuO_70_ZnO_30._ This catalyst shows the best properties for HER within the tested catalysts. Contrary, the catalyst CuO_50_ZnO_50_ shows a decrease in the current with a behaviour very close to ZnO, the catalyst with the lowest currents for HER. When NO_3_^-^ is added to the electrolyte (Fig. 3b, Fig. S6b, Supplementary Data 1,2), a general increase in the current density is observed for all the catalysts suggesting that the reduction of NO_3_^−^ is taking place. The addition of CO_2_ leads to a further increase in current, except for ZnO (Figs. 3c, S6c, Supplementary Data 1,2). Although the absolute current densities are higher for CuO_70_ZnO_30_, CuO_50_ZnO_50_ is the catalyst that shows the highest relative increase from below −0.1 mA/cm^2^ to −0.35 mA/cm^2^ in the presence of NO_3_^−^ and CO_2_ (Fig. S6, Supplementary Data 2).

It is important to note that at the electrode potentials of interest, the metal oxides might be reduced, as observed previously for CuO in other reactions^20^. However, recent studies have shown that the presence of Zn may stabilise some oxidised phases of Cu (Cu^δ+^)^37^. Clarification on this matter would require using in situ and operando techniques. This discussion is out of the scope of this manuscript, and to avoid misconceptions, the catalysts are always referred to as metal oxides.

**Fig. 4:**
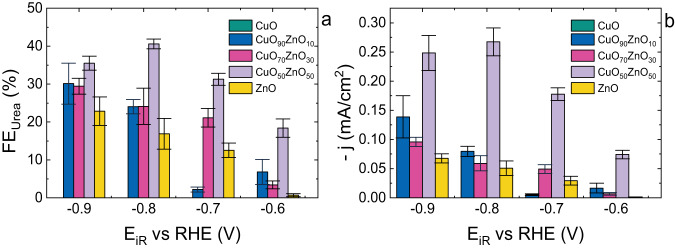
Electrocatalytic measurements for urea electrosynthesis. **a** Faradaic efficiency between −0.6 and −0.9 V vs RHE in 0.1 M Na_2_SO_4_ and 0.1 M NaNO_3_ with CO_2_, (**b**) Partial current density to urea between −0.6 and −0.9 V vs RHE in 0.1 M Na_2_SO_4_ and 0.1 M NaNO_3_ with CO_2_. The error bars display the average error obtained between three measurements.

The activity of the catalysts towards urea electrosynthesis was evaluated using chronoamperometry at fixed potentials. The catalysts were tested in an H-type electrochemical cell at mild conditions in 0.1 M Na_2_SO_4_ supporting electrolyte with 0.1 M NaNO_3_ saturated with CO_2_. The chronoamperometry experiments were conducted at potentials between −0.6 and −0.9 V vs RHE (iR corrected) with 0.1 V intervals. The obtained results, Faradaic efficiency (FE) and partial current density (j), were measured after 30 min of electrolysis and are shown in Fig. 4 (Supplementary Data 1). In all cases, except when CuO was used as the electrocatalyst, urea was produced from CO_2_ and NO_3_^-^ reduction. Although previous studies have reported the formation of urea on Cu electrodes^12,13^, it was not observed in our experiments. This may be explained by the possibility of lower activity with CuO compared to other Cu-based catalysts with different starting oxidation states, such as Cu or Cu_2_O. The implementation of different electrocatalytic set ups, or due to low produced amounts of urea that fall under the detection limits of the quantification method used. It should be mentioned that the detection of urea measured at potentials less negative than −0.7 V vs RHE has a high uncertainty due to very low current densities. For the ZnO catalyst, urea was observed starting from −0.7 V vs RHE and reaching up to 23% FE at –0.9 V vs RHE (Fig. 4a, Supplementary Data 1). These results agree with the results from Shibata et al. ^38,39^. It should be noted that in their work, the electrode used was Zn and not ZnO. Therefore, a direct comparison of the materials cannot be realised due to their different oxidation states. Nonetheless, when compared with oxygen vacancy-rich ZnO porous nanosheets^9^, the ZnO nanoparticles showed similar efficiencies. All the CuO_*x*_ZnO_*y*_ catalysts exhibited higher FE than ZnO, and the maximum FE was obtained at −0.8 V vs RHE for all the bimetallic catalysts. Within the studied catalysts, CuO_50_ZnO_50_ showed the highest efficiency of 41% at −0.8 V vs RHE (Fig. 4a, Supplementary Data 1). The FE decreased for potentials more negative than −0.8 V vs RHE, most likely due to the increase in the competing HER and NO_3_^-^ reduction to ammonia^20^. A similar trend to the FE was obtained when evaluating the partial current densities towards urea (Fig. 4b, Supplementary Data 1). All the catalysts showed an increase in partial current density starting from −0.6 V until −0.8 V vs RHE. At potentials more negative than −0.8 V vs RHE, all the activities decreased. The CuO_50_ZnO_50_ showed the highest partial current density within all the studied catalysts reaching the value of −0.27 mA/cm^2^ at −0.8 V vs RHE (Fig. 4b, Supplementary Data 1). Additionally, a clear dependence of the activity on the relative composition of the catalysts was observed (Figs. 4b, S7, Supplementary Data 1,2). This is likely related to electronic effects caused by the doping of Cu and Zn on the opposite oxides, as suggested by the increased lattice spacing (Fig. S2).

SEM analysis was performed for all the catalysts after reduction to evaluate the final morphology of the samples (Fig. S8). The CuO showed the formation of larger agglomerates with a spherical shape while ZnO showed a morphology closer to the one observed for the bimetallic catalysts prior to the reduction (Fig. 2a). This indicates a possible fragmentation of the larger particles that were initially observed for ZnO before the reduction reaction (Figs. 1a, 2a, Supplementary Data 1). All the CuO_*x*_ZnO_*y*_ catalysts exhibited some degree of particle segregation after reduction (Figure S8) compared to their initial morphology (Fig. 2a). However, an effect of the Cu/Zn ratio was evident since the CuO_50_ZnO_50_ sample showed larger agglomerates compared to the two other examined ratios. Lastly, the composition of the CuO_50_ZnO_50_ sample was tested with SEM-EDX (Fig. S9) after 2 h of electroreduction and a higher Cu/Zn ratio was found, showing the instability of Zn over prolonged electrolysis and its potential dissolution.

## Conclusions

In conclusion, the bimetallic system of CuO_*x*_ZnO_*y*_ was methodically studied by varying the CuO and ZnO ratios. These materials were used as electrocatalysts for the electrosynthesis of urea from CO_2_ and NO_3_^−^. The Faradaic efficiency and partial current density for urea over all the CuO_*x*_ZnO_y_ compositions tested were higher than those of ZnO or CuO. The Faradaic efficiency to urea reached 41% at −0.8 V vs RHE with the CuO_50_ZnO_50_. These results place this catalyst amongst the most efficient reported for urea electrosynthesis from CO_2_ and NO_3_^−^ (Table S2, Fig. S10). The activity dependence on the relative composition of the catalysts was attributed to possible electronic effects caused by the doping of Cu and Zn on the opposite oxides. The selectivity dependence on the ratios of CuO and ZnO shows that those should not be neglected when studying bimetallic catalysts for complex reactions such as urea electrosynthesis. To our awareness, this is the first publication that explores ratios of bimetallic catalysts for urea synthesis from CO_2_ and NO_3_^−^. Further characterisation of the materials and product analysis with in situ techniques is needed to understand the effect of Zn on the catalytic activity and mechanism. More work on these topics is in progress to better understand the mechanism of this process and the increased activity of CuO_*x*_ZnO_*y*_ materials.

## Methods

### Catalyst preparation

#### Flame spray pyrolysis synthesis

FSP is a single-step preparation method fitted for scale-up. The basic principle of FSP synthesis lies in injecting metal-containing liquid precursors into a methane-oxygen flame. The high temperature allows for the formation of metal oxides and limits sintering due to the fast evaporation of the solution droplets. The FSP synthesis was performed with a commercial TETHIS NPS10 set-up (Fig. S1). A precursor solution was prepared by dissolving the desired amounts of Cu(NO_3_)_2_·3H_2_O and/or Zn(NO_3_)_2_·6H_2_O salts in absolute ethanol to a total concentration of 0.15 M. For the CuO_x_ZnO_y_ catalysts, the Zn molarity was varied to target a particular Zn loading. The transparent precursor solution was transferred into the syringe of the FSP set-up. The solution was injected in the nozzle at a flow rate of 5.0 mL min^−1^. The flame was fed a 1.5 L min^−1^ methane flow and 3.0 L min^−1^ oxygen flow. The additional oxygen dispersion flow was 5.0 L min^−1^, giving rise to an overpressure of ~2.5 bar. During pyrolysis, the nanoparticles were formed and deposited on a quartz filter positioned in the set-up’s upper part. The powder was collected from the filter and sieved with a 500 µm sieve to remove filter residues. The powders were then directly used without further drying or calcination since the high temperature of FSP synthesis fully decomposes the precursors.

#### Electrode preparation

The electrodes were prepared by drop-casting the desired catalyst ink on a carbon paper substrate (Sigracet 22bb, Fuelcellstore). For the CuO and CuO_*x*_ZnO_*y*_ electrodes, an ink of 5 mg catalyst powder was mixed with 1 ml of ethanol and 50 μl of Nafion. The ink was then sonicated for 20 min and drop casted on 5 cm^2^ of carbon paper, resulting in a loading of 1 mg/cm^2^. For the ink containing ZnO the ethanol was replaced with 500 μl of ultrapure water and 500 μl of isopropanol to obtain a better catalyst dispersion.

### Catalyst characterisation

#### X-ray diffraction

The x-ray diffraction patterns were acquired with a Bruker D2 Phaser diffractometer equipped with a Cu Ka radiation source. An increment size of 0.02° and a time of 0.5 s were used. It is noted that the diffraction peak location was based on the diffraction patterns of CuO (PDF-00-005-0661) and ZnO (PDF-00-065-0726) of the International Centre for Diffraction Data.

#### Wide-angle X-ray scattering

The wide-angle X-ray scattering measurements were performed at the beamline ID31 of the ESRF synchrotron. An incident photon energy of 75 keV (0.0165 nm) and a Pilatus CdTe 2 M detector were used in a Debye-Scherrer geometry.

The reflection position was transferred from *q* (reciprocal) to 2θ (real) space based on Eq. (1).

1 $$\left|{{q}}\right|=\left(4\times \frac{{{{{{\rm{\pi }}}}}}}{{{{{{\rm{\lambda }}}}}}}\right)\times \sin \left(\frac{2{{{{{\rm{\theta }}}}}}}{2}\right)$$

where λ is the wavelength.

The assignment of the reflections was based on existing literature and evaluated using Bragg’s law, Eq. (2).

2 $${{n}}{{{{{\rm{\lambda }}}}}}=2{{{{{\rm{d}}}}}}\times \sin {{{{{\rm{\theta }}}}}}$$

where *n* is the order of reflection, λ is the wavelength and *d* is the interplanar spacing.

#### X-ray photoelectron spectrometry

The X-ray photoelectron spectra were collected with a K-Alpha ultra-high vacuum X-ray photoelectron spectrometer by ThermoFisher Scientific equipped with a monochromatic aluminium anode (Kα = 1486.68 eV, 72 W) X-ray source with a spot size of 400 μm and a 180° double focusing hemispherical analyser with a 128-channel detector. The samples were measured at a pass energy of 50 eV. The survey spectra determined the sample composition. The elements detected were copper, zinc, oxygen, and carbon. The charging states of the elements of interest were analysed with core-level lines C 1 s, O 1 s, Cu 2p, Cu LMM, Zn 2p and Zn LMM. The spectra were processed using the CasaXPS software. The binding energy scale was adjusted by setting the binding energy of the C 1 s to 284.8 eV. The XPS spectra were fitted using a Shirley background, and the curve fitting was carried out with a Gaussian function GL(30) for Cu 2p and Laurenzian asymmetric function LA(1.4,2,2) for Zn 2p. In detail, the Cu 2p_3/2_ spectra (RSF = 18.1471) were fitted by setting a peak area constraint for Cu^2+^ equal to 1.36 times larger than the shake-up peak. An additional peak was fitted to account for the asymmetry of Cu^2+^. The area constraint applied was the same, the position constraint used was equal to 1.4 eV higher than the main Cu^2+^ peak, and the FWHM was set to Cu^2+^ +1.42. The Zn 2p spectra (RSF = 31.8614) were fitted with a position constraint equal to 23.1 eV between the Zn 2p_2/3_ and Zn 2p_1/3_.

#### Transmission electron microscopy

Transmission electron microscopy was performed with an FEI Tecnai (Sphera) microscope operating at an acceleration voltage of 200 kV. The catalyst particles were dispersed by ultrasonication in ethanol and afterwards deposited on a Cu grid. The particle sizes were measured using ImageJ software.

#### Scanning electron microscopy (SEM) with energy-dispersive X-ray spectroscopy (EDX)

The scanning electron micrographs were measured with an FEI QUANTA 3D microscope equipped with an energy dispersive detector (EDS) and electron-backscatter diffraction (EBDS) detector. The energy dispersive X-ray spectrometry was performed with an accelerating voltage of 15 kV and a beam current of 4 nA. The surface morphology of the materials was obtained at the secondary electrons acquisition mode, using an acceleration voltage of 20 kV and a beam current of 26.7pA.

#### Inductively coupled plasma optical emission spectrometry (ICP-OES)

The elemental analysis of copper present in the electrolyte solutions was performed on an EOP ICP optical emission spectrometer (Spectroblue) with axial plasma viewing, equipped with a free-running 27.12 MHz generator (1400 W).

#### Electrochemical active surface area measurements

The electrochemical active surface area of the electrodes was determined by measuring their double-layer capacitance. The capacitance value of each electrode was acquired by cyclic voltammograms at different scan rates in the potential window of where no Faradaic processes occur. The capacitance is equal to the linear regression slope of the charging currents against the scan rates at a specific potential. The capacitance of each electrode was calculated based on the linear regression plot of the current vs the scan rates (Fig. S11, Supplementary Data 2). Each electrochemical active area was then calculated (Table S3) by dividing the obtained capacitance with the specific capacitance (Cs) equal to 40 μF cm^−2^ for CuO and CuO_*x*_ZnO_*y*_ electrodes^40^. The specific capacitance (Cs) equal to 30μF cm^−2^ was used for the ZnO.

#### Electrochemical measurements

All the electrochemical cells and glassware used for the electrochemical measurements were thoroughly cleaned before each experiment to avoid organic and inorganic contaminations. The decomposition of organic contaminations was accomplished by storing the glassware overnight in an aqueous solution of 1 g L^–1^ KMnO_4_ (98%, Alfa Aesar) and 0.5 M H_2_SO_4_ (95–97%, Merck). The following day, the KMnO_4_ solution was drained, and any remaining KMnO_4_ was removed with 10% H_2_O_2_ (33%, VWR). Finally, the glassware was boiled three times in ultrapure type 1 water (18.2 M*Ω* cm, Purelab flex, ELGA LabWater).

A homemade peak H-cell with a fixed electrode distance was used for all the electrochemical experiments. The two compartments were separated by an anionic membrane (FAA-3-pk-130) supplied by FuelCellstore. The membrane was activated prior to the measurements by immersion in a 0.5 M KOH solution for 24 h. Following this, the membrane was rinsed and stored in ultrapure water. An annealed platinum foil (1 cm^2^) was used as a counter electrode, and a leakless Ag/AgCl as a reference (ATO72-1, Edaq). The 0.1 M Na_2_SO_4_ (Emsure, Merck) and 0.1 M Na_2_SO_4_ and 0.1 M NaNO_3_ (Emsure, Merck) solutions (pH=7.6) were prepared with ultrapure type I water. The solution was pre-saturated with CO_2_ prior to the measurement (pH=5.7). To avoid possible dissolution during the reaction and a correct charge calculation, the catalysts were reduced at 0.25 V vs RHE before each experiment to ensure a full reduction before the applied reaction potential^41^. The electrode potentials measured on the Ag/AgCl scale were converted to the reversible hydrogen electrode (RHE) scale using the following equation:

3 $${{{E}}}_{{{{{{\rm{RHE}}}}}}}=0.197+{{{E}}}_{{{{{{\rm{Ag}}}}}}/{{{{{{\rm{AgC}}}}}}}{{{{{\rm{l}}}}}}}+0.059{{{{{\rm{V}}}}}}\times {{{{{\rm{pH}}}}}}$$

All the experiments were performed at room temperature. The potentiostats used for the experiments were an Autolab PGSTAT204 and an Autolab AUT302 (Metrohm) equipped with the NOVA 2.1 software.

### Product analysis

Ultraviolet–visible spectroscopy was used to quantify urea after its decomposition to ammonia. The decomposition of urea into carbon dioxide and ammonia (${({{{{{{\rm{NH}}}}}}}_{2})}_{2}{{{{{\rm{CO}}}}}}\mathop{\longrightarrow }\limits^{{{{{\mathrm{urease}}}}}}{{{{{{\rm{CO}}}}}}}_{2}+2{{{{{{\rm{NH}}}}}}}_{3}$) was realised by the enzyme urease and the moles of urea were calculated according to the Eq. (4):

4 $${{{m}}}_{{{{{{\rm{urea}}}}}}}=\frac{{{{m}}}_{{{{{{\mathrm{urease}}}}}}}-{{{m}}}_{{{{{{\rm{ammonia}}}}}}}}{2}$$

where *m*_urease_ are the total moles of ammonia in the sample measured after the urea decomposition and *m*_ammonia_ the total moles of ammonia measured before the urea decomposition, the ammonia concentrations were measured before and after the enzyme addition based on the salicylate method^42^. To decompose urea, a urease solution was prepared by diluting one urease tablet (Tablets, Fisher Scientific) to 10 ml of ultrapure water.

Before adding the enzyme solution, the pH of the catholyte sample was tested to ensure that the pH was at a range where the enzyme was active (pH range: 6–9). Subsequently, 0.3 ml of urease solution were added to 2.7 ml of catholyte sample and heated at 40 °C for 40 min. The pH was tested again and adjusted with sodium hydroxide to reach 12 before proceeding with the salicylate method^42^. In detail, 2 ml of the catholyte sample solution were mixed with 1 M sodium hydroxide solution (50%, EMSURE, Merck) containing 5 wt% salicylic acid (≥ 99%, GPR RECTAPUR, VWR Chemicals) and 5 wt% sodium citrate (trisodium citrate dihydrate ≥ 99%, Thermo Scientific). Subsequently, 1 mL of 0.05 M sodium hypochlorite solution (14% Cl_2_ in aqueous solution, GPR RECTAPUR, VWR Chemicals) and 0.2 mL of 1 wt% sodium nitroprusside dihydrate aqueous solution (99.0–102.0%, AnalaR NORMAPUR, VWR Chemicals) were added. The samples were prepared in amber plastic bottles and kept in the dark at room temperature for 45 minutes to allow for colour development and limit the photodecomposition of the reagent. The mixtures were measured in plastic cuvettes (10 mm, PMMA, VWR Chemicals) between 800 and 450 nm. The quantification of ammonia before and after urea decomposition was calculated based on the absorption intensities at a wavelength of 655 nm according to the calibration curves for ammonia and ammonia from decomposed urea, respectively (Figs. S12, S13, Supplementary Data 2). The catholyte samples were diluted to a fitting concentration to ensure that the absorbance fell within the linear range of the calibration curve.

### Faradaic efficiency and partial current density calculation

The FE for the urea production from the nitrate and carbon dioxide reduction was calculated based on Eq. (5):

5 $${{{{{\rm{FE}}}}}}=\frac{{{n}}\times {{F}}\times {{C}}}{{{Q}}}$$

where *n* is the number of electrons involved in the formation of urea (16 e^−^), *F* is the Faraday’s constant (96485 C mol^−1^), *C* is the product concentration in moles, and *Q *(*C*) is the total charge that passed through the electrode during the electrolysis.

The partial current density for urea was calculated based on Eq. (6):

6 $${{j}}=\frac{{{Q}}\times {{{{{\rm{FE}}}}}}}{{{t}}\times {{A}}}$$

where *Q *(*C*) is the total charge passed through the electrode during the electrolysis, FE is the Faradaic efficiency, *t* (s) is the total measurement time (30 min), and *A* is the electrochemically active surface area of the electrode (cm^2^)

All the reported Faradaic efficiencies and current densities were determined based on three measurements. The error bars represent the standard deviation between the three measurements.

### Supplementary information

Suplementary Information

Description of supplementary files

Supplementary Data 1

Supplementary Data 2

## Data Availability

The authors declare that the data supporting the findings of this study are available within the paper Supplementary Information and Supplementary Data file 1 and Supplementary Data file 2. All other data are available from the corresponding author on reasonable request.
