# Supplementary material for: Carbon dioxide and nitrate co-electroreduction to urea on CuOxZnOy
Source: Commun Chem. 2023 Sep 19;6:199. doi: 10.1038/s42004-023-01001-5 (PMC10509248; doi:10.1038/s42004-023-01001-5)
Supplement: Supplementary file 1 — Suplementary Information [file 42004_2023_1001_MOESM1_ESM.pdf]

## Supplementary information

# Carbon dioxide and nitrate co-electroreduction to urea on $CuO_xZnO_y$


Dimitra Anastasiadou[1], Bianca Ligt[1], Yunyang He[1], Rim C.J. van de Poll[1], Jérôme F.M. Simons[1], Marta Costa Figueiredo[1,2]*

[1] Department of Chemical Engineering and Chemistry Department, Eindhoven University of Technology, PO Box 513, Eindhoven 5600 MB, the Netherlands. E-mail: m.c.costa.figueiredo@tue.nl

[2] Eindhoven Institute of Renewable Energy Systems (EIRES), Eindhoven University of Technology, PO Box 513, Eindhoven 5600 MB, the Netherlands.


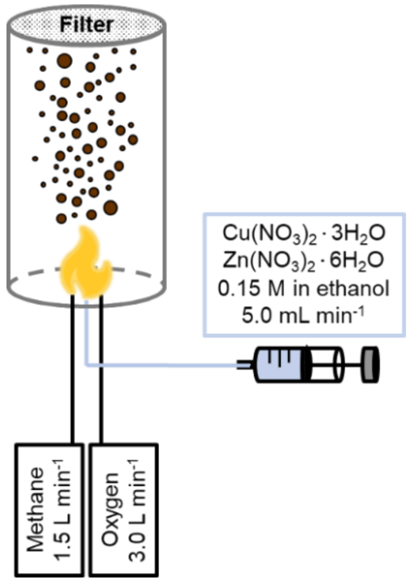


**Figure S1. Flame spray pyrolysis synthesis set-up.**

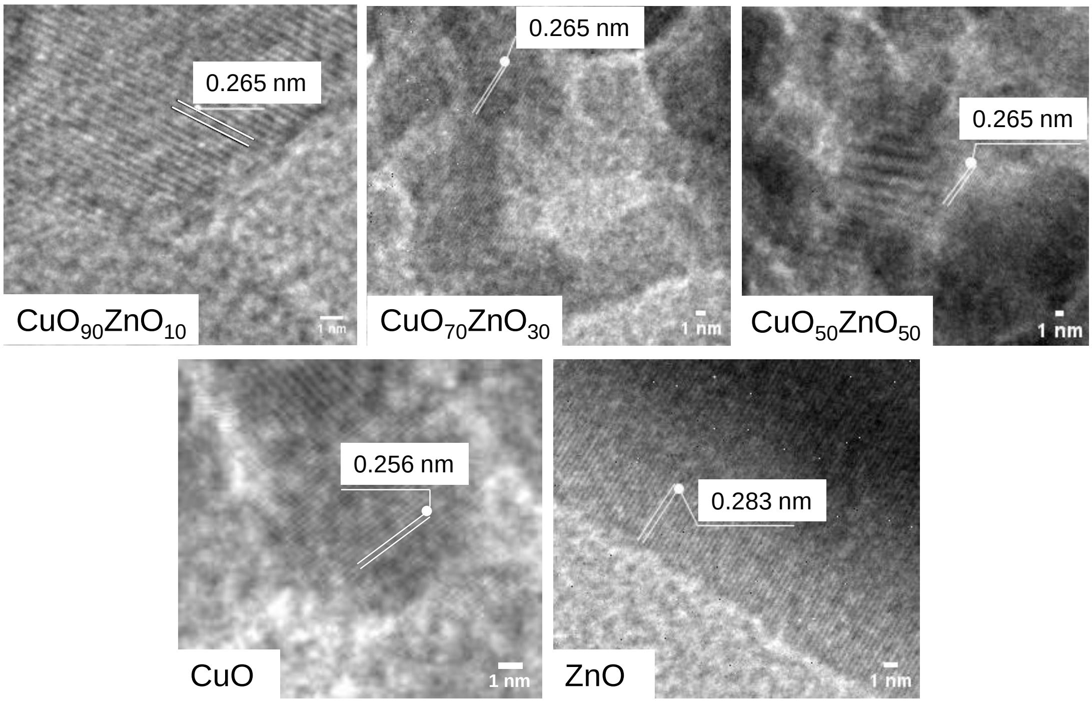


**Figure S2. TEM images of the $CuO_xZnO_y$ catalysts after synthesis.**

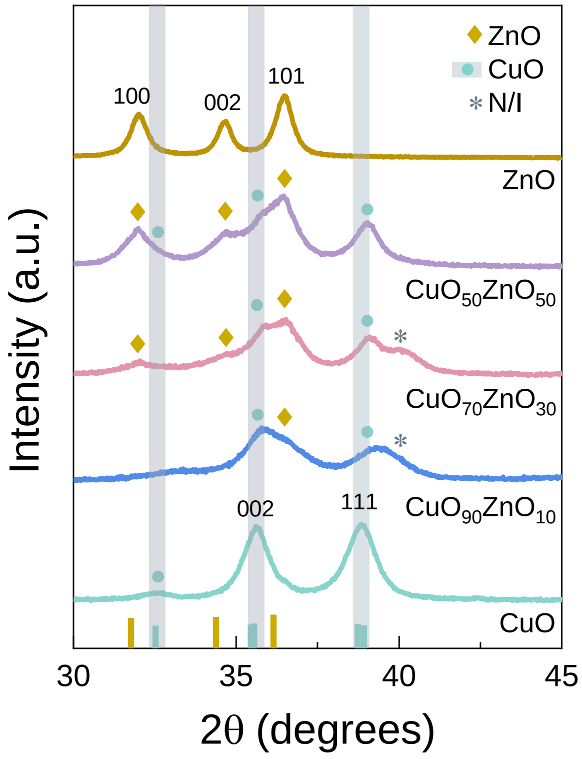


**Figure S3. XRD patterns between 30- 45 degrees, of the $CuO_xZnO_y$ catalysts after synthesis, PDF-00-065-0726, (—) PDF-00-005-0661(—).**

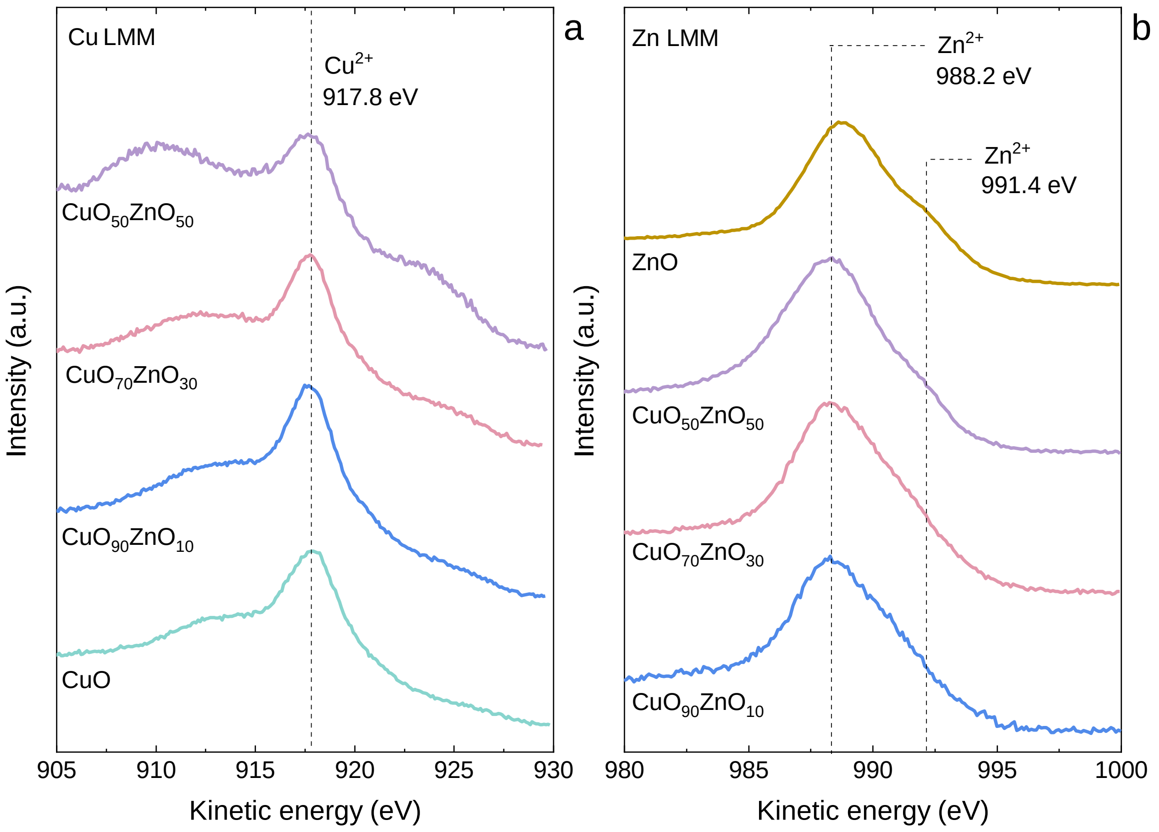


**Figure S4. XPS auger spectra, a) Cu LMM and b) Zn LMM of the catalysts after synthesis.**

**Table S1. Elemental composition**

| | XPS | | ICP | | SEM-EDX | |
|---|---|---|---|---|---|---|
| Sample | Cu at% | Zn at% | Cu at% | Zn at% | Cu at% | Zn at% |
| $CuO_{90}ZnO_{10}$ | 84 | 16 | 89.0 | 11.0 | 88.3 | 11.7 |
| $CuO_{70}ZnO_{30}$ | 66 | 34 | 69.4 | 30.6 | 66.8 | 33.2 |
| $CuO_{50}ZnO_{50}$ | 44 | 56 | 49.9 | 50.1 | 49.8 | 50.2 |

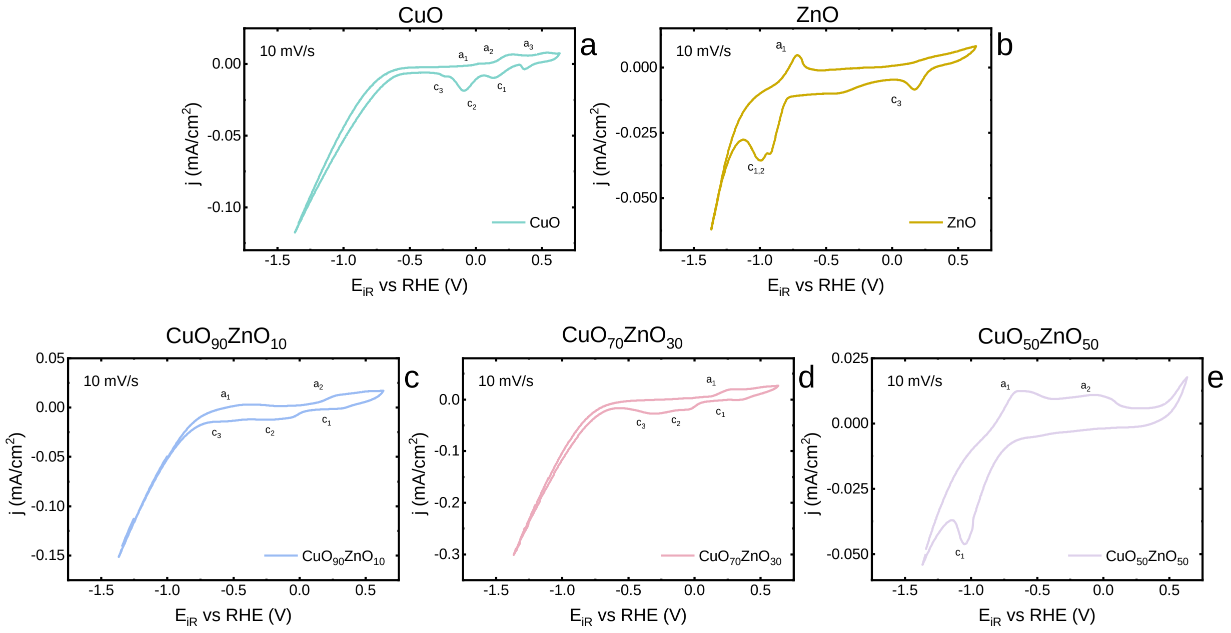


**Figure S5. Cyclic voltammograms in 0.1 M $Na_2SO_4$, a) CuO, b) ZnO, c) $CuO_{90}ZnO_{10}$, d) $CuO_{70}ZnO_{30}$, e) $CuO_{50}ZnO_{50}$. Scan rate: 10 mV/s**

Figure S5a shows the CV of CuO, the peaks labelled $a_{1-3}$ and $c_{1-3}$ are a result from the oxidation of Cu to $Cu^{2+}$/ $Cu^{+}$ species and their reduction to Cu, respectively. In Figure S5b the redox features of ZnO are visible around -1.5 V vs Ag/AgCl. At the cathodic scan of ZnO there are two overlapping but well-defined reductive peaks $c_{1,2}$ and broad oxidation peak $a_1$ compounded from two overlapping anodic peaks. According to previous studies the number of anodic and cathodic peaks as well as their shape is dependent on the surface.[1] The redox features present at the CVs of the $CuO_xZnO_y$ electrodes revealed the presence of both Cu and Zn. Cu characteristic redox features were predominant at the electrodes with higher Cu loading while the Zn redox features are not distinct. On the other hand, $CuO_{50}ZnO_{50}$ prevalently shows the ZnO redox features (Figure S5e, $a_1$, $c_1$) and a broad oxidation peak corresponding to the oxidation of Cu (Figure S5e, $a_2$) with no Cu reduction peak is present.

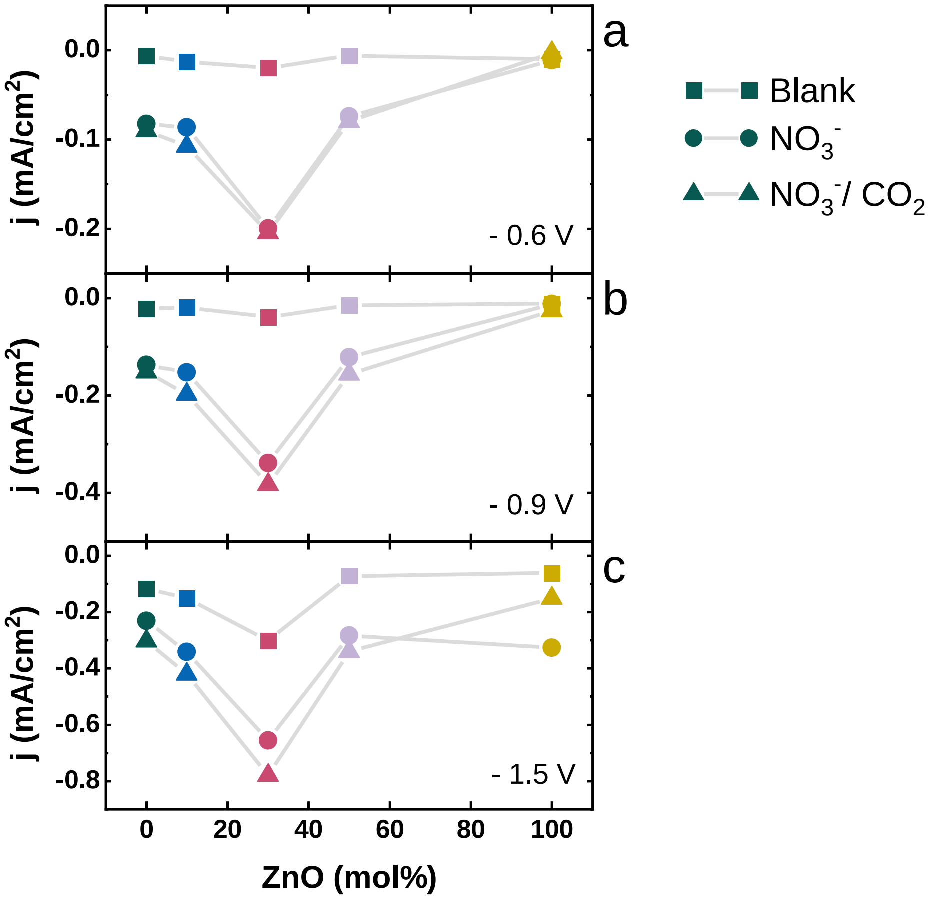


**Figure S6. Maximum current density reached per sample based on CVs a) -0.6 V, b) -0.9 V and c) –1.5 V vs RHE in 0.1 M $Na_2SO_4$ (blank), in 0.1 M $Na_2SO_4$ and 0.1 M $NaNO_3$ and in 0.1 M $Na_2SO_4$ and 0.1 M $NaNO_3$ with $CO_2$.**

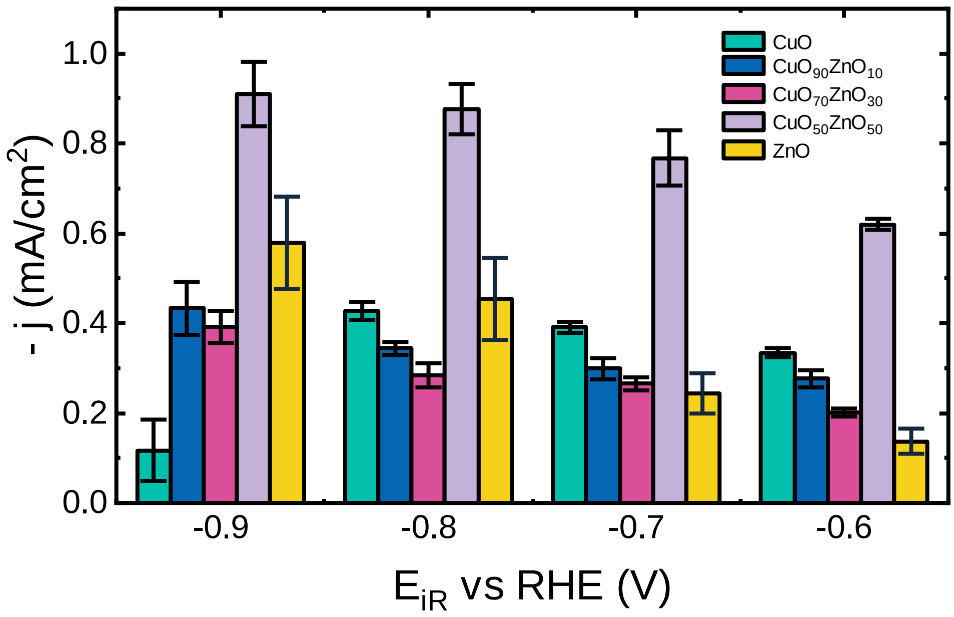


**Figure S7. Total current density per sample in 0.1 M $Na_2SO_4$ and 0.1 M $NaNO_3$ with $CO_2$. The error bars display the average error obtained between three measurements.**

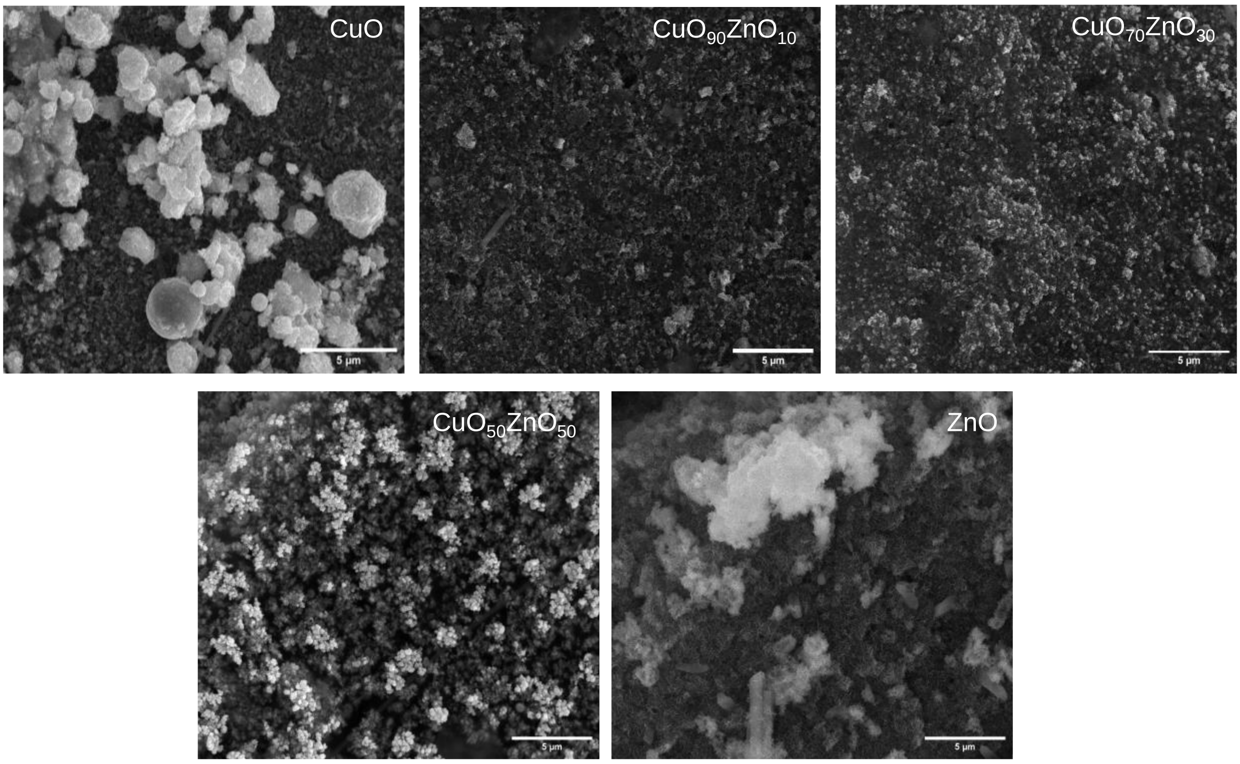


**Figure S8. SEM of the catalysts after 2h of reduction in 0.1 M $Na_2SO_4$ and 0.1 M $NaNO_3$ with $CO_2$.**

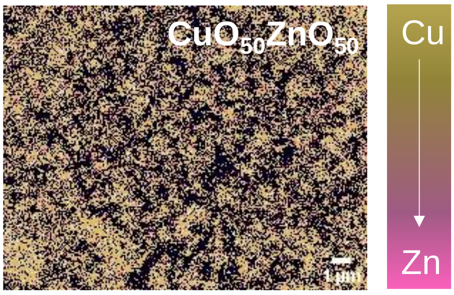


**Figure S9. SEM- EDX of the $CuO_{50}ZnO_{50}$ catalyst after reaction for 2h at -0.8 V vs RHE in 0.1 M $Na_2SO_4$ and 0.1 M $NaNO_3$ with $CO_2$.**

The elemental composition of the sample based on the SEM-EDX results after 2 hours of electroreduction is 94.1 at% of Cu and 5.9 at% of Zn.

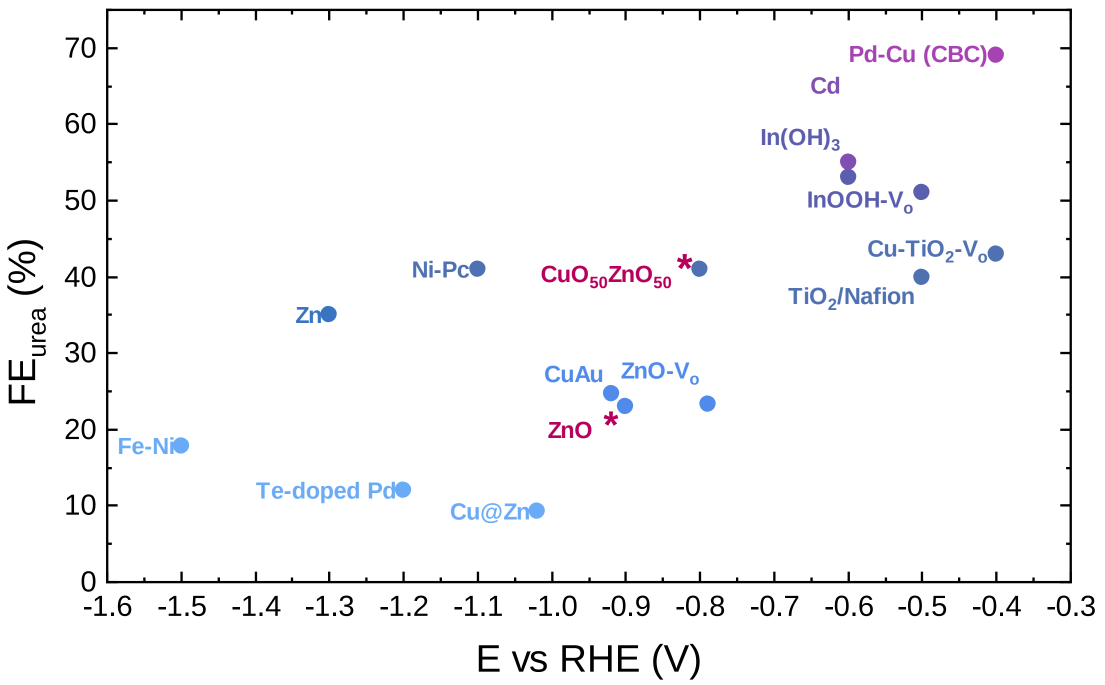


**Figure S10. Faradaic efficiency for urea from $NO_{3/2}^-$ and $CO_2$ with different electrocatalytic systems. (* This work).**

**Table S2. Electrocatalytic systems for the synthesis of urea from $CO_2$ and nitrate/nitrite.**

| Catalyst | Reactants | Supporting electrolyte | Product | FE (%) | Current Density (mA/cm$^2$) | Potential (V vs RHE) | Ref. |
|---|---|---|---|---|---|---|---|
| CuAu | $CO_2$, $NO_2^-$ | 0.5 M $KHCO_3$ | $CO(NH_2)_2$ | 24.7 | ca. -20 | -0.92 | 2 |
| Cu@Zn | $CO_2$, $NO_3^-$ | 0.2 M $KHCO_3$ | $CO(NH_2)_2$ | 9.28 | -3.13 ($j_{urea}$) | -1.02 | 3 |
| Pd-Cu /carbonized bacterial cellulose (CBC) | $CO_2$, $NO_3^-$ | 0.05 M $KNO_3$ | $CO(NH_2)_2$ | 69.1 | - | -0.4 | 4 |
| Cu-$TiO_2$ nanotubes-$V_o$ | $CO_2$, $NO_3^-$ | 0.2 M $KHCO_3$ | $CO(NH_2)_2$ | 43 | -3.2 ($j_{urea}$) | -0.4 | 5 |
| $TiO_2$/Nafion | $CO_2$, $NO_3^-$ | 0.1 M $KNO_3$ | $CO(NH_2)_2$ | 40 | ca. -20 | -0.5 | 6 |
| nano Fe-$TiO_3$ | $CO_2$, $NO_2^-$ | 1M $NaHCO_3$ | $CO(NH_2)_2$ | - | -0.8 ($j_{urea}$) | - | 7 |
| Fe-Ni | $CO_2$, $NO_3^-$ | 0.1 M $KHCO_3$ | $CO(NH_2)_2$ | 17.8 | ca. -42 | -1.5 | 8 |
| Ni-Pc | $CO_2$, $NO_3^-$ | 0.2 M $KHCO_3$ | $CO(NH_2)_2$ | 41 | -11 | -1.1 | 9 |
| Te-doped Pd | $CO_2$, $NO_2^-$ | 0.1 M $KHCO_3$ | $CO(NH_2)_2$ | 12 | -0.8 ($j_{urea}$) | -1.2 | 10 |
| ZnO-$V_o$ | $CO_2$, $NO_2^-$ | 0.2 M $NaHCO_3$ | $CO(NH_2)_2$ | 23.26 | ca. -25 | -0.79 | 11 |
| Zn | $CO_2$, $NO_3^-$ | 0.2 M $KHCO_3$ | $CO(NH_2)_2$ | 35 | - | -1.3 | 12 |
| $CeO_2$-$V_o$ | $CO_2$, $NO_3^-$ | 0.1 M $KHCO_3$ | $CO(NH_2)_2$ | - | - | -1.6 | 13 |
| InOOH-$V_o$ | $CO_2$, $NO_3^-$ | 0.1 M $KNO_3$ | $CO(NH_2)_2$ | 51 | -1 | -0.5 | 14 |
| $In(OH)_3$ | $CO_2$, $NO_3^-$ | 0.1 M $KNO_3$ | $CO(NH_2)_2$ | 53 | -0.3 | -0.6 | 15 |
| Cd | $CO_2$, $NO_2^-$ | 0.2 M $KHCO_3$ | $CO(NH_2)_2$ | 55 | - | -0.6 | 12 |
| $CuO_{50}ZnO_{50}$ | $CO_2$, $NO_3^-$ | 0.5 M $Na_2SO_4$ | $CO(NH_2)_2$ | 41 | -0.27 ($j_{urea}$) | -0.8 | This work |
| ZnO | $CO_2$, $NO_3^-$ | 0.5 M $Na_2SO_4$ | $CO(NH_2)_2$ | 23 | -0.06 ($j_{urea}$) | -0.9 | This work |

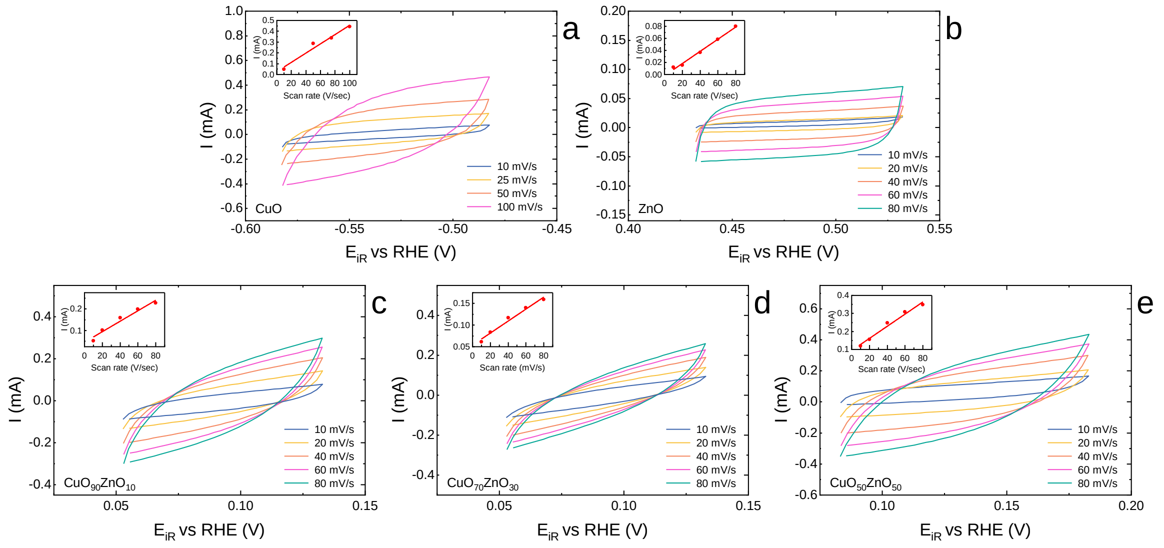


**Figure S11. Electrochemical double layer capacitance measurements a) CuO, b) ZnO, c) $CuO_{90}ZnO_{10}$, d) $CuO_{70}ZnO_{30}$, e) $CuO_{50}ZnO_{50}$.**

**Table S3. Electrochemical active surface area (ECSA) values.**

| | **CuO** | **ZnO** | **$CuO_{90}ZnO_{10}$** | **$CuO_{70}ZnO_{30}$** | **$CuO_{50}ZnO_{50}$** |
|---|---|---|---|---|---|
| **ECSA ($cm^2$)** | 107.13 | 50.70 | 60.63 | 34.70 | 84.33 |

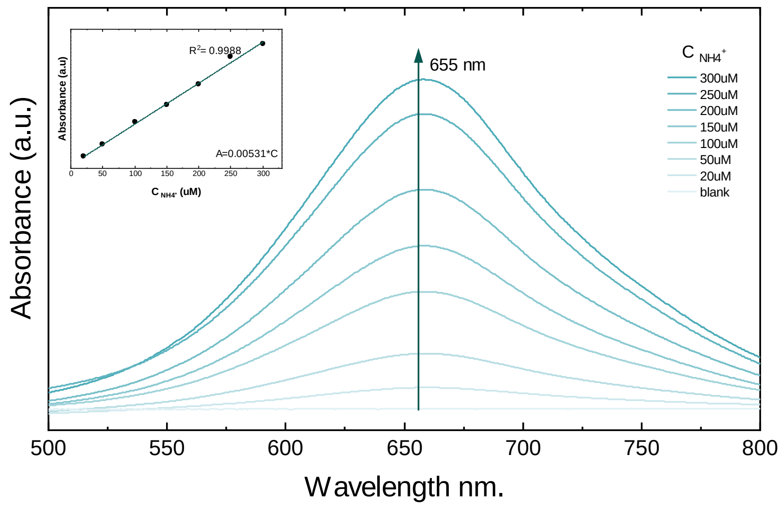


**Figure S12. UV-Vis calibration curve for $NH_4^+$ quantification based on the salicylate method.**

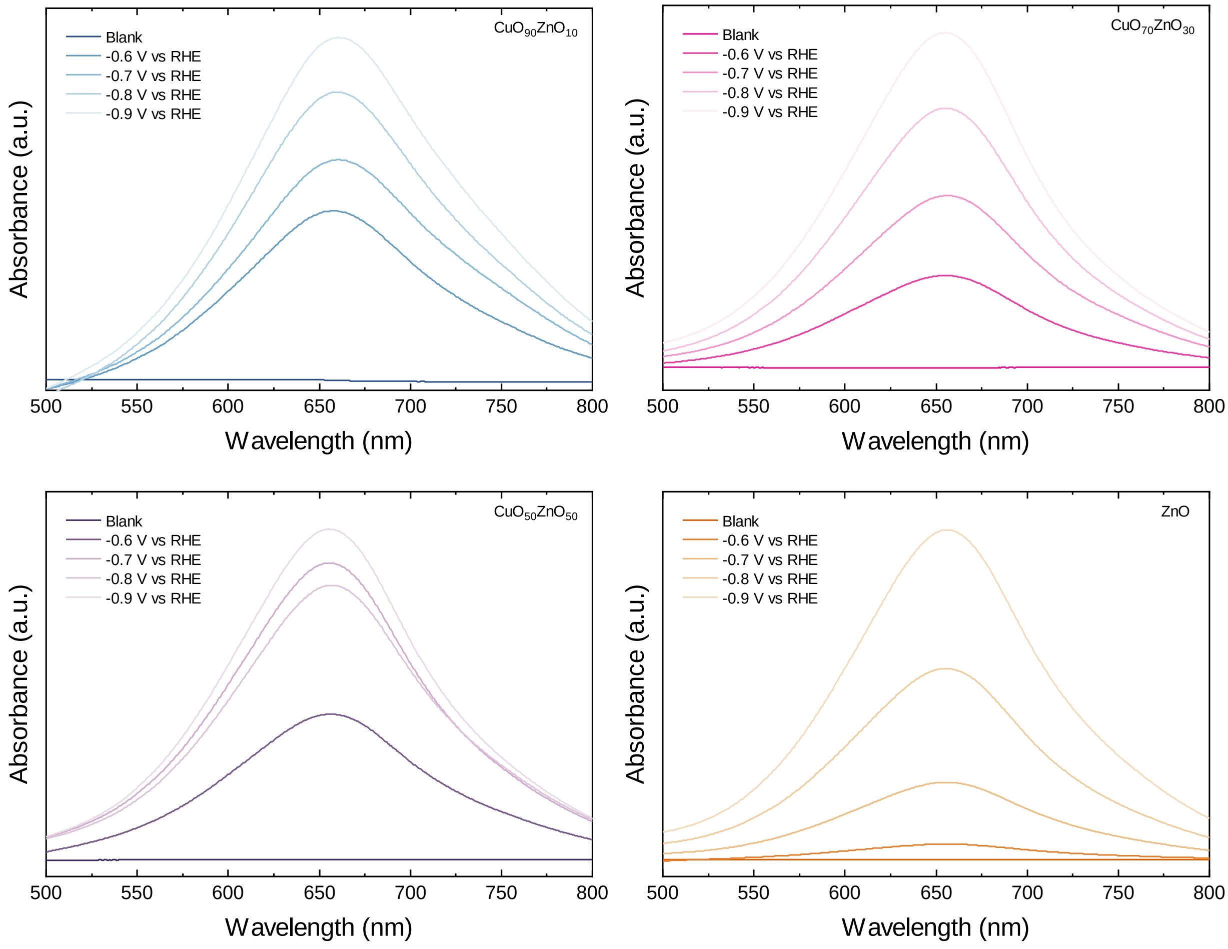


**Figure S13. UV-Vis spectra for urea quantification based on the salicylate method.**

## Supplementary references


1. Sulciute, A.; Nishimura, K.; Gilshtein, E.; Cesano, F.; Viscardi, G.; Nasibulin, A. G.; Ohno, Y.; Rackauskas, S. ZnO Nanostructures Application in Electrochemistry: Influence of Morphology. *Journal of Physical Chemistry C* **2021**, *125* (2), 1472–1482. https://doi.org/10.1021/acs.jpcc.0c08459.
2. Tao, Z.; Wu, Y.; Wu, Z.; Shang, B.; Rooney, C.; Wang, H. Cascade Electrocatalytic Reduction of Carbon Dioxide and Nitrate to Ethylamine. *Journal of Energy Chemistry* **2022**, *65*, 367–370. https://doi.org/10.1016/j.jechem.2021.06.007.
3. Liu, S.; Yin, S.; Wang, Z.; Xu, Y.; Li, X.; Wang, L.; Wang, H. AuCu Nanofibers for Electrosynthesis of Urea from Carbon Dioxide and Nitrite. *Cell Rep Phys Sci* **2022**, *3* (5), 100869. https://doi.org/10.1016/j.xcrp.2022.100869.
4. Meng, N.; Ma, X.; Wang, C.; Wang, Y.; Yang, R.; Shao, J.; Huang, Y.; Xu, Y.; Zhang, B.; Yu, Y. Oxide-Derived Core–Shell Cu@Zn Nanowires for Urea Electrosynthesis from Carbon Dioxide and Nitrate in Water. *ACS Nano* **2022**, *16* (6), 9095–9104. https://doi.org/10.1021/acsnano.2c01177.
5. Zhang, S.; Geng, J.; Zhao, Z.; Jin, M.; Li, W.; Ye, Y.; Li, K.; Wang, G.; Zhang, Y.; Yin, H.; Zhang, H.; Zhao, H. High-Efficiency Electrosynthesis of Urea over Bacterial Cellulose Regulated Pd–Cu Bimetallic Catalyst. *EES Catalysis* **2023**, *1* (1), 45–53. https://doi.org/10.1039/d2ey00038e.
6. Cao, N.; Quan, Y.; Guan, A.; Yang, C.; Ji, Y.; Zhang, L.; Zheng, G. Oxygen Vacancies Enhanced Cooperative Electrocatalytic Reduction of Carbon Dioxide and Nitrite Ions to Urea. *J Colloid Interface Sci* **2020**, *577*, 109–114. https://doi.org/10.1016/j.jcis.2020.05.014.
7. Saravanakumar, D.; Song, J.; Lee, S.; Hur, N. H.; Shin, W. Electrocatalytic Conversion of Carbon Dioxide and Nitrate Ions to Urea by a Titania-Nafion Composite Electrode. *ChemSusChem* **2017**, *10* (20), 3999–4003. https://doi.org/10.1002/cssc.201701448.
8. Siva, P.; Prabu, P.; Selvam, M.; Karthik, S.; Rajendran, V. Electrocatalytic Conversion of Carbon Dioxide to Urea on Nano-FeTiO3 Surface. *Ionics (Kiel)* **2017**, *23* (7), 1871–1878. https://doi.org/10.1007/s11581-017-1985-1.
9. Zhang, X.; Zhu, X.; Bo, S.; Chen, C.; Qiu, M.; Wei, X.; He, N.; Xie, C.; Chen, W.; Zheng, J.; Chen, P.; Jiang, S. P.; Li, Y.; Liu, Q.; Wang, S. Identifying and Tailoring C–N Coupling Site for Efficient Urea Synthesis over Diatomic Fe–Ni Catalyst. *Nat Commun* **2022**, *13* (1), 5337. https://doi.org/10.1038/s41467-022-33066-6.
10. Shibata, M.; Furuya, N. Simultaneous Reduction of Carbon Dioxide and Nitrate Ions at Gas-Diffusion Electrodes with Various Metallophthalocyanine Catalysts. *Electrochim Acta* **2003**, *48* (25–26), 3953–3958. https://doi.org/10.1016/S0013-4686(03)00534-6.
11. Feng, Y.; Yang, H.; Zhang, Y.; Huang, X.; Li, L.; Cheng, T.; Shao, Q. Te-Doped Pd Nanocrystal for Electrochemical Urea Production by Efficiently Coupling Carbon Dioxide Reduction with Nitrite Reduction. *Nano Lett* **2020**, *20* (11), 8282–8289. https://doi.org/10.1021/acs.nanolett.0c03400.
12. Meng, N.; Huang, Y.; Liu, Y.; Yu, Y.; Zhang, B. Electrosynthesis of Urea from Nitrite and CO2 over Oxygen Vacancy-Rich ZnO Porous Nanosheets. *Cell Rep Phys Sci* **2021**, *2* (3), 100378. https://doi.org/10.1016/j.xcrp.2021.100378.
13. Shibata, M.; Yoshida, K.; Furuya, N. Electrochemical Synthesis of Urea at Gas-Diffusion Electrodes: III. Simultaneous Reduction of Carbon Dioxide and Nitrite Ions with Various Metal Catalysts. *J Electrochem Soc* **1998**, *145* (2), 595–600. https://doi.org/10.1149/1.1838309.
14. Wei, X.; Wen, X.; Liu, Y.; Chen, C.; Xie, C.; Wang, D.; Qiu, M.; He, N.; Zhou, P.; Chen, W.; Cheng, J.; Lin, H.; Jia, J.; Fu, X. Z.; Wang, S. Oxygen Vacancy-Mediated Selective C-N Coupling toward Electrocatalytic Urea Synthesis. *J Am Chem Soc* **2022**, *144* (26), 11530–11535. https://doi.org/10.1021/jacs.2c03452.
15. Lv, C.; Lee, C.; Zhong, L.; Liu, H.; Liu, J.; Yang, L.; Yan, C.; Yu, W.; Hng, H. H.; Qi, Z.; Song, L.; Li, S.; Loh, K. P.; Yan, Q.; Yu, G. A Defect Engineered Electrocatalyst That Promotes High-Efficiency Urea Synthesis under Ambient Conditions. *ACS Nano* **2022**, *16*, 8222. https://doi.org/10.1021/acsnano.2c01956.
16. Lv, C.; Zhong, L.; Liu, H.; Fang, Z.; Yan, C.; Chen, M.; Kong, Y.; Lee, C.; Liu, D.; Li, S.; Liu, J.; Song, L.; Chen, G.; Yan, Q.; Yu, G. Selective Electrocatalytic Synthesis of Urea with Nitrate and Carbon Dioxide. *Nat Sustain* **2021**, *4* (10), 868–876. https://doi.org/10.1038/s41893-021-00741-3.
17. Wu, Y.; Jiang, Z.; Lin, Z.; Liang, Y.; Wang, H. Direct Electrosynthesis of Methylamine from Carbon Dioxide and Nitrate. *Nat Sustain* **2021**, *4* (8), 725–730. https://doi.org/10.1038/s41893-021-00705-7.
