# Supplementary material for: Carbon dioxide and nitrate co-electroreduction to urea on CuOxZnOy
Source: Commun Chem. 2023 Sep 19;6:199. doi: 10.1038/s42004-023-01001-5 (PMC10509248; doi:10.1038/s42004-023-01001-5)
Supplement: Supplementary file 2 — Description of supplementary files [file 42004_2023_1001_MOESM2_ESM.pdf]

Description of Additional Supplementary Files

File name: Supplementary Data 1

Description: Dataset of the figures in the manuscript

File name: Supplementary Data 2

Description: Dataset of the figures in supplementary information
